# Real world efficacy and safety of the advanced hybrid closed-loop system MiniMed 780G (SmartGuard) in children under 7 years of age

**DOI:** 10.3389/fmed.2024.1465800

**Published:** 2025-01-06

**Authors:** Sara López-López, Cristina Díaz-Martín, Inés García-de Pablo, María Teresa Ovejero-Garcia, María Beatriz Garnier-Rodríguez, Ruth Molina-Suárez, Cristina Ontoria-Betancort, Blanca Sáez-Gallego, Ángela Domínguez-García, Sofía Quinteiro-González, Lourdes Travieso-Suárez, María Fátima Cabrera-Guedes, Yeray Nóvoa-Medina

**Affiliations:** ^1^Complejo Hospitalario Universitario Insular Materno Infantil de Canarias, Las Palmas de Gran Canaria, Gran Canaria, Spain; ^2^Hospital Universitario Nuestra Señora de la Candelaria, Santa Cruz de Tenerife, Tenerife, Spain; ^3^Hospital Universitario de Canarias, La Laguna, Tenerife, Spain; ^4^Instituto Universitario de Investigaciones Biomédicas y Sanitarias, Universidad de Las Palmas de Gran Canaria, Las Palmas de Gran Canaria, Spain; ^5^Asociación Canaria para la Investigación Pediátrica (ACIP Canarias), Las Palmas de Gran Canaria, Spain

**Keywords:** type 1 diabetes, closed-loop, children, automated insulin delivery, treatment

## Abstract

**Objective:**

To evaluate the safety and efficacy of the Medtronic 780G SmartGuard™ AID system in children under 7 years of age with type 1 diabetes (T1D).

**Methods:**

Retrospective analysis of data from children living with T1D under 7 years of age using the MiniMed 780G™ across three pediatric endocrinology units in the Canary Islands. Metabolic control parameters were analyzed from 14 days of pretreatment to 12 months of follow-up.

**Results:**

The study included 61 children under 7 years of age, 35 in Group 1 and 26 in Group 2. In Group 1, there was a significant increase in time in range (TIR) (13%, *p* = 0,000), along with a significant decrease in time above range (TAR) (7% for TAR1 and 3% for TAR2; *p* = 0,000). These improvements persisted for up to 1 year of follow-up. In Group 2, there was a significant increase in the TIR (7%; *p* = 0,000) and a significant decrease in the TAR (7%; *p* = 0,000 for TAR1 and 6.5%; *p* = 0,001 for TAR2). These improvements persisted for up to 6.5 months of follow-up. No significant changes were observed in the time below range (TBR) or variation coefficient (CV) in either group. No events of severe hypoglycemia or diabetic ketoacidosis occurred. Efficacy and safety were maintained in children with a TDD <8 units/day.

**Conclusion:**

The use of the Medtronic 780G™ SmartGuard™ system in children under 7 years of age with T1D is effective and safe, with benefits persisting for up to 6–12 months. The safety profile is maintained in children receiving a TDD <8 units/day.

## Introduction

Type 1 diabetes (T1D) is a complex disease that affects a growing proportion of the population worldwide (1). Poor disease control results in chronic hyperglycemia, leading to severe complications, including retinopathy, nephropathy, neuropathy, heart disease and peripheral vascular system damage. It also impairs immune function and increases oxidative stress, exacerbating acute illnesses. These complications underscore the critical importance of maintaining good metabolic control to prevent long-term damage and improve quality of life for children with T1D.

Over time, recommended glycemic targets have become more rigorous, with recent technological advances such as continuous glucose monitoring (CGM), continuous subcutaneous insulin infusion (CSII) and automated insulin delivery (AID) facilitating metabolic control while decreasing the risk of hypoglycemia (2). Current international guidelines for pediatric T1D recommend maintaining HbA1c levels below 7% or achieving more than 70% time in range (TIR) (interstitial glucose concentration between 70 and 180 mg/dL) (2, 3). Some national guidelines, such as the UK National Institute for Health and Care Excellence (NICE), propose even lower targets (6.5%) (4).

Achieving these strict targets is particularly challenging in younger children due to various factors, including unpredictable eating patterns and activities, frequent illnesses, minimal doses and fear of hypoglycemia. However, the advent of AID systems has revolutionized T1D management, offering new possibilities for improved glycemic outcomes without increasing the risk of hypoglycemia (2, 5, 6). Furthermore, CSII has been recommended as the treatment of choice in children younger than 7 years of age (7), and current evidence supports its use, where available, in all children with T1D due to the benefits it provides in metabolic management (8) and quality of life for both children and their families (9, 10).

There are currently different brands of CSII that interact with CGM and algorithms to allow AID. Most of them are only approved for children older than 6–7 years of age. Medtronic is one of them. Its current AID system (MiniMed 780G™) is an advanced hybrid closed-loop insulin delivery system that uses SmartGuard™ technology to automatically adjust insulin delivery based on continuous glucose monitoring (CGM) data. It uses the Guardian sensor 4 to measure interstitial glucose levels every 5 min. Depending on the glucose values and using the SmartGuard™ algorithm, the system adjusts insulin delivery to maintain glucose levels in the desired range. The system can deliver correction boluses or suspend insulin delivery depending on the glucose concentration. It is currently only approved for children 7 years of age and older who use a TDD of more than 8 units (11) after the FDA reviewed the data published by Bergental et al. in 2021 evaluating the safety and efficacy of the MiniMed 780G™ system in adolescents and young adults (12). However, a few studies have successfully proven its safety in younger children (13–16).

Given the published safety data and the difficulty in controlling our younger study participants, we used the MiniMed 780G™ with SmartGuard™ mode under close supervision in this age group. This article aims to describe the safety and efficacy of the Medtronic MiniMed 780G™ SmartGuard™ AID system in children younger than 7 years of age. By examining the performance of this advanced technology in very young children, we hope to shed light on its potential to address the specific needs of this age group and contribute to improved management strategies for our youngest children living with T1D.

## Materials and methods

### Design and population

This was a descriptive, observational, retrospective study. Children living with T1D were recruited from the three main pediatric endocrinology units in the Canary Islands: Hospital Universitario de Canarias (Santa Cruz de Tenerife), Hospital Universitario Nuestra Señora de la Candelaria (Santa Cruz de Tenerife) and Complejo Hospitalario Universitario Insular Materno Infantil (Gran Canaria).

The inclusion criteria included children who were diagnosed with T1D according to the American Diabetes Association (ADA) criteria (2), who were under 7 years of age, who had any duration of their disease, who were using the MiniMed 780G™ system at the time of the analysis, either directly in SmartGuard™ mode or with initial time in predictive low glucose suspend and who subsequently, still with age < 7 year, changed to AutoMode. All study participants were on MDI therapy prior to the initiation of Medtronic MiniMed 780G™ system. Exclusion criteria: Age 7 years or older at the start of SmartGuard™ mode, or inability to access data through the CareLink platform.

There are two groups of children in the study. Group 1 included participants living with T1D in whom CSII was initiated in manual mode (predictive low glucose suspend), with a subsequent change to SmartGuard™ mode. Group 2 included participants in whom the SmartGuard™ mode was initiated at the beginning of CSII use (directly from MDI treatment).

The Minimed780G™ system was chosen for our population based on its approval and coverage by our healthcare system during that period.

### Data collection

The data were collected retrospectively from the charts of children treated in the aforementioned pediatric endocrinology units. This review included a description of the characteristics of children living with T1D aged under 7 years and treated with the MiniMed 780G™ system, as well as follow-up data related to metabolic control. We analyzed data from 14 days before treatment modification, until 1–3–6-12 months for both groups.

The parameters studied for both groups were TIR (70–180 mg/dL), time above range (TAR) 1 and 2 (TAR1: 181–250 mg/dL and TAR2: >250 mg/dL), time below range (TBR) 1 and 2 (TBR1: 54–69 mg/dL and TBR2: <54 mg/dL), glucose management indicator (GMI), average glucose, total daily insulin dose (TDD) and coefficient of variation (CV).

### Statistical analysis

SPSS version 21 (IBM SPSS Statistics for Windows, Armonk, NY, United States) was used for statistical analysis of the data. The Shapiro–Wilk test was used to verify the normality of the distribution. For descriptive statistics, the mean and standard deviation were determined for normally distributed quantitative variables, while the median and interquartile range were calculated for nonnormally distributed variables. Qualitative variables are described as frequencies.

Repeated-measures ANOVA was conducted to assess significant changes in normally distributed variables over time. This method was chosen to account for the within-subject correlation and to identify any overall temporal effects. *Post hoc* analyses were performed using the Bonferroni correction to determine the specific time points responsible for significant changes in trends, ensuring the robustness of the findings by controlling for multiple comparisons. For nonnormally distributed variables, the Friedman test was used to evaluate the presence of changes in the studied variable over time. *Post hoc* analysis was performed via pairwise comparisons using the Wilcoxon signed-rank test with Bonferroni correction.

For group 1, initially, the analysis was performed between the moment when study participants were in the predictive low glucose suspension/manual mode (14 days before initiation of SmartGuard™) and 1, 3 and 6 months after initiation of SmartGuard™. For group 2, the analysis was performed between the moment when children were on multiple doses of insulin (MDI) and 1 and 3 months after initiation of SmartGuard™ to maximize the sample size. Additionally, two-way ANOVA was employed in group 1 to analyze differences between the periods when study participants were on the MDI and in the low-glucose suspension/manual mode. Subsequently, follow-up data up to 1 year and 6.5 months were included for groups 1 and 2 respectively, to provide a more comprehensive evaluation of long-term effects.

To evaluate the impact of having a dose lower than 8 units/day on the efficacy and safety of SmartGuard™ (TIR and TBR, respectively), we used the Mann–Whitney U test to compare the independent means (children with ≥8 units/day vs. children with <8 units/day) at 14 days after the initiation of Smartguard™ in group 1 and 14 and 45 days after the initiation of SmartGuard™ in group 2. We chose those moments in time because they were the only ones with 3 or more study participants receiving <8 units/day.

## Results

Sixty-one children living with T1D aged less than 7 years were included in the study. 35 for group 1 and 26 for group 2. The results were similar in both groups:

### From MDI to manual mode, and then SmartGuard™

**Figure 1:**
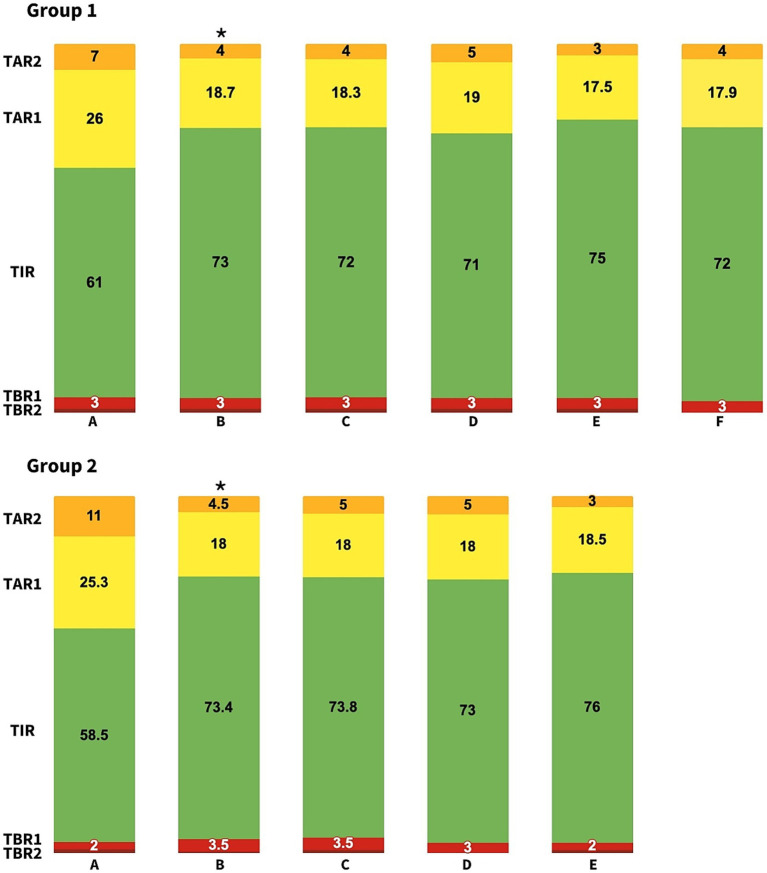
Changes in control parameters during follow up for Group 1 and Group 2 of patients at 14 days before SmartGuardTM initiation **(A)**, 14 days after SmartGuardTM initiation **(B)**, 45 days **(C)**, 3.5 months **(D)**, 6.5 months **(E)**, and 1 year **(F)**. TIR, Time in range; TAR, Time above range; TBR, Time below range. *Statistically significant changes compared to baseline.

The descriptive statistics are summarized in Table 1. Table 2 presents the values of the analyzed variables over time. Repeated-measures ANOVA (and the Friedman test when appropriate) revealed a significant increase in the TIR [χ^2^(4) = 35.6, *p* < 0.000], along with a decrease in the GMI, mean glucose [*F*(4) = 11.7, *p* < 0.000], TAR1 [*F*(4) = 17.9, *p* < 0.000] and TAR2 [χ^2^(4) = 23.5, *p* < 0.000] following the initiation of SmartGuard™ (Figure 1). No significant changes were observed in the TBR1 [χ^2^(4) = 8.3, *p* = 0.08], TBR2 [χ^2^(4) = 1.9, *p* = 0.79] or the CV [*F*(4) = 0.8, *p* = 0.51] (Supplementary Table S2). Significant differences persisted after Bonferroni corrections between the manual mode and initiation of SmartGuard™ (Supplementary Table S1). No significant differences were found among the other time points (*p* > 0.05). Supplementary Table S2 summarizes the test statistics for both ANOVA and the Friedman test.

**Table 1:** Summary of descriptive statistics.

	Group 1	Group 2
Sample size	35	26
Sex (% female)	33.3%	53.8%
Antibody positive (%)	97.1%	84.6%
Age onset (years. Mean ± SD)	2.4 ± 1.2	2.5 ± 1.5
Years with T1D at CSII start (median ± IQ range)	0.8 ± 1.7	
Years with T1D at CSII start (mean ± SD)		1.5 ± 1.3
Years with T1D at data collection (mean ± SD)	2.9 ± 1.9	2.2 ± 1.7
Age starts manual mode (years. Mean ± SD)	3.4 ± 1.5	
Age starts SmartGuard™ (years. Mean ± SD)	3.8 ± 1.5	4.2 ± 1.7

1. Group 1: Initial CSII in manual mode and posterior SmartGuard™. 2. Group 2: Direct SmartGuard™ mode.

**Table 2:** Changes in control parameters over time after CSII and SmartGuard™ initiation.

	MDI	Manual mode	SmartG 14d	SmartG 45d	SmartG 3.5 m	SmartG 6.5 m	SmartG 12 m
Group 1							
TIR	60.0 (23.5)	61.0 (16.5)	73.0 (12) ƚ	72.0 (13.5)	71.0 (16.5)	75.0 (17)	72.0 (15.5)
TAR1*	26.6 ± 16	26.0 ± 8.5	18.7 ± 6.2 ƚ	18.3 ± 6.2	18.7 ± 7.1	17.5 ± 5.6	17.9 ± 5.8
TAR2	5.5 (17.5)	7.0 (9.5)	4.0 (6.5) ƚ	4.0 (7)	5.0 (6)	3.0 (7)	4.0 (6.25)
TBR1	3.5 (6.25)	3.0 (3)	3.0 (2.5)	3.0 (2)	3.0 (2.5)	3.0 (1.5)	3.0 (2)
TBR2	1.0 (1.75)	1.0 (1)	1.0 (1)	1.0 (1)	1.0 (1)	1.0 (1)	0 (1)
Mean glucose*	158.8 ± 24	160.0 ± 17.1	144.4 ± 17 ƚ	147.8 ± 12.5	147.8 ± 20.1	143.0 ± 11.5	146.6 ± 12
GMI	7.0 (0.77)	7.4 (0.75)	6.8 (0.4) ƚ	7 (0.45)	6.7 (0.55)	6.9 (0.4)	6.75 (0.52)
CV*	37.0 ± 5.9	36.5 ± 4.9	37.8 ± 5	37.5 ± 4.4	37.0 ± 4.7	37.5 ± 5.7	37.3 ± 5.7
Group 2							
TIR*	58.5 ± 17	---	73.4 ± 7.3 ƚ	73.8 ± 7	72.9 ± 7.4	76.07 ± 5.2	---
TAR1*	25.3 ± 10.3	---	18.05 ± 5 ƚ	17.95 ± 6.4	18.4 ± 6.4	18.5 ± 4.9	---
TAR2	11.0 (16.75)	---	4.5 (6) ƚ	5.0 (4.2)	5.0 (6.5)	3.0 (3)	---
TBR1	2.0 (2.75)	---	3.5 (3)	3.5 (3)	3.0 (1)	2.0 (3)	---
TBR2	1.0 (1)	---	0.5 (1)	1.0 (1)	0 (1)	1.0 (1)	---
Mean glucose	170 (43.5)	---	147.5 (18.7) ƚ	144.0 (16)	140.0 (16.5)	142.0 (19)	---
GMI*	7.2 (0.9)	---	6.8 (0.3) ƚ	6.7 (0.2)	6.9 (0.6)	6.7 (0.3)	---
CV*	36.7 ± 6.1	---	37.05 ± 4.4	36.7 ± 4.6	37.3 ± 5	---	---

TAR, Time above range; TBR, Time below range; GMI, Glucose management indicator; CV, Variation coefficient. ^*^Mean ± SD. The rest are expressed as Median (Interquartile range). ^Ƚ^Appearance of statistically significant changes.

Subsequent analysis up to 1 year of follow-up demonstrated that these trends persisted, with maintained significant improvements in the TIR and reductions in the TAR1 and TAR2. Additionally, two-way ANOVA (or the Friedman test when appropriate) was conducted to compare the effects of MDI and manual CSII. This analysis revealed no significant differences in the values between the MDI and manual mode CSII periods.

Regarding the total daily insulin dose (TDD), we did not find any significant change either from the time of low glucose suspension/manual mode to 6 m after SmartGuard™ initiation [*F*(4) = 2.5; *p* = 0.13] or from MDI mode to 1 year after SmartGuard™ initiation [*F*(6) = 2.6; *p* = 0.23]. Two children had a TDD of less than 8 units/day when starting the SmartGuard™ system. The lowest dose was 5.1 units/day. A low TDD had no impact on the efficacy (TIR, *p* = 0.54) or safety profile of AID (TBR1 and TBR2, *p* = 0.38 and 0.14, respectively).

### From MDI to SmartGuard™ directly

The descriptive statistics are summarized in Table 1. Table 2 presents the values of the analyzed variables over time. Repeated-measures ANOVA (and the Friedman test when appropriate) revealed a significant increase in TIR [*F*(4) = 12.6, *p* < 0.000], along with a decrease in TAR1 [*F*(3) = 10.4, *p* < 0.000], TAR2 [χ^2^(4) = 16.4, *p* = 0.001], GMI [χ^2^(4) = 14.8, *p* = 0.005] and mean glucose [χ^2^(4) = 14.8, *p* = 0.005] following the initiation of SmartGuard™ (Figure 1). No significant changes were observed in the TBR1 [χ^2^(4) = 3.4, *p* = 0.49], TBR2 [χ^2^(4) = 4, *p* = 0.4] or the CV [*F*(4) = 0.44, *p* = 0.78] (Supplementary Table S2). *Post hoc* comparisons using the Bonferroni correction (and pairwise comparisons using the Wilcoxon signed-rank test with Bonferroni correction when appropriate) indicated that the changes in TIR, GMI, mean glucose and TAR were specifically due to differences between the MDI and the initiation of SmartGuard™ (Supplementary Table S1). No significant differences were found among the other time points (*p* > 0.05).

Subsequent analysis up to 6.5 months of follow-up demonstrated that these trends persisted, with maintained significant improvements in the TIR, mean glucose, GMI and TAR.

Regarding the TDD, we found significant changes between 14 days after the initiation of SmartGuard™ and 6 months later (*F*(3) = 5.5; *p* = 0.03). The difference is due only to changes between 14 days after initiation and the 6-month time point (not with any of the in-between moments). No significant changes were detected between the MDI and 14 days after the initiation of SmartGuard™ [probably due to the low number of subjects (14) and large standard deviation (SD) (9.3)] [*F*(1) = 3.2; *p* = 0.097]. Four of our participants started SmartGuard™ with a TDD of less than 8 units, with that number increasing to 6 after 3 months and decreasing thereafter. The lowest dose was 5.8 units/day. We found no difference in the efficacy or safety profile of the SmartGuard™ mode in children receiving lower doses compared to those receiving a TDD >8 units/day for 14 days (TIR, *p* = 0.18; TBR1 and TBR2 = 0.47 and 0.41, respectively) or 45 days after the initiation of the SmartGuard™ mode (TIR, *p* = 0.65; TBR1 and TBR2 = 1 and 0.82, respectively).

No study participants presented episodes of severe hypoglycemia or ketoacidosis after the initiation of AID.

## Discussion

In this article, we evaluated the safety and efficacy of the MiniMed 780G™ system in children younger than 7 years of age diagnosed with T1D. With 61 children living with T1D and a follow-up of up to 12 months, our study is one of the largest and longest follow-up studies to date and one of the few to include children receiving low doses (<8 units/day). Our results revealed improvements in metabolic parameters in both groups, with improved average glucose levels and increased TIR up to 6–12 months. Additionally, safety is highlighted by the increased TIR due to decreased TAR without increased TBR, meaning that the risk of hypoglycemia is not increased. We also evaluated the efficacy and safety of its use in participants with low doses (< 8 units/day), and our results support its use.

CamAPS FX™, developed at the University of Cambridge and integrated with compatible devices for automated insulin dosing, along with Omnipod 5 in the US, are the only AID system that currently have approval for children younger than 6 years of age (17). However, other systems, such as the Tandem t:slim X2™ insulin pump with Control-IQ technology™ integrated with the Dexcom sensor (18, 19), the Omnipod 5™ Automated Insulin Delivery System integrated with the Dexcom G6™ (20) or, prior to the use of the MiniMed 780G™, the MiniMed 670G™ system (21–24), have been proven to be efficacious and safe in children younger than 6–7 years of age. These studies show an increase in the TIR of approximately 8–13%, which is similar to our 13–15% increase.

The MiniMed 780G™ has also been used in young children, and its safety and efficacy have been proven, albeit with low numbers of study participants (13–15). Abraham et al. recently reported a short follow-up of 10 children using the MiniMed 780G™ system, with safe and efficacious outcomes (16). Pulkkinen et al. described their results after using the same system in 35 children younger than 7 years of age after a 12-week follow-up period (13). All of their study participants had a dose higher than 8 units/day. They reported significant improvements in glucose values without an increase in hypoglycemia. They argued that the lack of change in the TBR could be due to the efficient pretrial treatment, given that most of their participants used either the predicted low glucose suspension or another HCL system, and only 3 of them used MDI prior to the introduction of the MiniMed 780G™ system. They recently reported persistence of their good results after 18 months follow up (15). In our sample, 34 children used MDI prior to using the HCL system, but we did not observe an increase in TBR, highlighting its safety. Although the sample size is relatively small, to our knowledge, it is the highest reported to date in this age group.

Tornese et al. reported a smaller number of children (12) with 1 year follow-up, and included children with doses lower than 8 units/day (14). They reported similar results, supporting the safety and efficacy of the MiniMed 780G™ system in young children. Their experience in children with the lowest TDD was similar to ours, reporting a safe and efficacious glucose profile when using the Smartguard™ mode.

It is worth mentioning that the improvement described during the follow-up appeared 2 weeks after the initiation of the Smartguard™ system and was maintained until the end of our follow-up. No further improvements were observed after those 2 weeks. Similar results were published by Tornese et al. (14), with improvements in the TIR after a short period. These improvements were maintained but did not further improve during the follow-up period. Similar results have been reported by other authors with follow-up for 1 (25) and 2 years (26), albeit in older children. We think this might be due to several reasons: first of all, our sample size might not be big enough to detect small differences. Also, the rapid improvement probably represents the immediate benefits of switching to an AID system, resulting from the algorithms ability to make frequent adjustments to insulin delivery. After those 2 weeks, the algorithm’s ability might have limitations due to its design, safety parameters to prevent hypoglycemia, system constraints (sensor accuracy, insulin absorption variability and speed of insulin action) and meal-related challenges (initial improvement might reflect better basal control, while meal-related changes in glycemia could remain a challenge).

Some of the limitations of our study are its retrospective nature and the lack of a control group. Additionally, even though the number of children included in the study is among the largest for this age group, as we mentioned before, it is still a small number. This is especially evident in the group with the lowest TDD (<8 units/day), with only 6 study participants. Also, adding the emerging metric time in tight range (TITR) would have given added value to our analysis (27).

Some of the strengths are the number of children included in the study (one of the largest to date), its long follow-up period and its multicenter nature, which limit the presence of bias.

In conclusion, in our young population with T1D, the use of the MiniMed 780G™ improves metabolic management compared to other insulin delivery methods, such as MDI or CSII, while maintaining a good safety profile. These improvements in metabolic control and safety are maintained in children with a low TDD (< 8 units/day). More studies including a larger number of participants with low doses (< 8 units/day) are needed to confirm the safety and efficacy of their use.

## Data Availability

The original contributions presented in the study are included in the article/Supplementary material, further inquiries can be directed to the corresponding author.
