# Supplementary material for: Real world efficacy and safety of the advanced hybrid closed-loop system MiniMed 780G (SmartGuard) in children under 7 years of age
Source: Front Med (Lausanne). 2025 Jan 6;11:1465800. doi: 10.3389/fmed.2024.1465800 (PMC11743179; doi:10.3389/fmed.2024.1465800)
Supplement: Supplementary file 1 [file Table_1.DOCX]

**Supplementary Table 1**. Post hoc comparisons using the Bonferroni correction

| **Comparison** | **MD** | **SE** | **p-value (Bonferronni)** |
| --- | --- | --- | --- |
| **TIR(1) - TIR(2)** | -10.3 | 2 | 0.000 |
| **TAR1(1) - TAR1(2)** | 7.3 | 1.4 | 0.000 |
| **TAR2(1) - TAR2(2)** | 3.7 | 1 | 0.011 |
| **TBR1(1) - TBR1(2)** | -0.7 | 0.3 | 0.32 |
| **TBR2(1) - TBR2(2)** | 0.036 | 0.2 | 1 |
| **VC(1) - VC(2)** | -1.3 | 0.7 | 1 |

**MD**: Mean Difference; **SE**: Standard Error; **TIR**: Time in Range;

**TAR**: Time Above Range; **TBR:** Time below Range; **VC**: Variation Coefficient

**Supplementary table 2**. Summary of analysis of temporal variation

| Source | SS | df | MS | F statistic | p-value | Partial η² | χ² | df | p-value |
| --- | --- | --- | --- | --- | --- | --- | --- | --- | --- |
| Group 1 |  |  |  |  |  |  |  |  |  |
| TIR | -- | -- | -- | -- | -- | -- | 35.6 | 4 | 0.000 |
| TAR1 | 1371.5 | 4 | 342.9 | 17.9 | 0.000 | 0.39 | -- | -- | -- |
| Linear | 791.2 | 1 | 791.2 | 51.7 | 0.000 | 0.65 | -- | -- | -- |
| TAR2 | -- | -- | -- | -- | -- | -- | 23.5 | 4 | 0.000 |
| TBR1 | -- | -- | -- | -- | -- | -- | 8.3 | 4 | 0.08 |
| TBR2 | -- | -- | -- | -- | -- | -- | 1.9 | 4 | 0.75 |
| Mean glucose | 5643.27 | 4 | 1410.8 | 11.7 | 0.000 | 0.294 | -- | -- | -- |
| Linear | 2924.9 | 1 | 2924.9 | 24.6 | 0.000 | 0.47 | -- | -- | -- |
| GMI | -- | -- | -- | -- | -- | -- |  |  |  |
| CV | 27.70 | 4 | 6.9 | .8 | 0.51 | 0.032 | -- | -- | -- |
| Linear | 4.6 | 1 | 4.6 | .3 | 0.58 | 0.013 | -- | -- | -- |
| Group 2 |  |  |  |  |  |  |  |  |  |
| TIR | 2125.7 | 4 | 531.4 | 12.6 | 0.000 | 0.48 | -- | -- | -- |
| Linear | 1131.6 | 1 | 1131.6 | 14.5 | 0.002 | 0.51 | -- | -- | -- |
| TAR1 | 722.355 | 3 | 240.785 | 10.461 | 0.000 | 0.37 | -- | -- | -- |
| Linear | 398.2 | 1 | 398.2 | 12.1 | 0.003 | 0.4 | -- | -- | -- |
| TAR2 | -- | -- | -- | -- | -- | -- | 16.4 | 4 | 0.001 |
| TBR1 | -- | -- | -- | -- | -- | -- | 3.4 | 4 | 0.49 |
| TBR2 | -- | -- | -- | -- | -- | -- | 4 | 4 | 0.4 |
| Mean glucose | -- | -- | -- | -- | -- | -- | 21.5 | 4 | 0.000 |
| GMI | -- | -- | -- | -- | -- | -- | 14.8 | 4 | 0.005 |
| CV | 24.7 | 4 | 6.18 | 0.44 | 0.78 | 0.03 | -- | -- | -- |
| Linear | 0.778 | 1 | 0.778 | 0.037 | 0.849 | 0.003 | -- | -- | -- |

**SS**: Sum of Squares; **df**: Degrees of freedom; **MS**: Mean Square; **F**: F statistic; **TAR**: Time Above Range; **TBR:** Time below Range; **CV**: Variation Coefficient
